# A Ploidy Difference Represents an Impassable Barrier for Hybridisation in Animals. Is There an Exception among Botiid Loaches (Teleostei: Botiidae)?

**DOI:** 10.1371/journal.pone.0159311

**Published:** 2016-07-21

**Authors:** Jörg Bohlen, Vendula Šlechtová, Vlastimil Šlechta, Vera Šlechtová, Alexandr Sember, Petr Ráb

**Affiliations:** Institute of Animal Physiology and Genetics AS CR, v.v.i., Rumburská 89, 277 21 Liběchov, Czech Republic; Virginia Commonwealth University, UNITED STATES

## Abstract

One of the most efficient mechanisms to keep animal lineages separate is a difference in ploidy level (number of whole genome copies), since hybrid offspring from parents with different ploidy level are functionally sterile. In the freshwater fish family Botiidae, ploidy difference has been held responsible for the separation of its two subfamilies, the evolutionary tetraploid Botiinae and the diploid Leptobotiinae. Diploid and tetraploid species coexist in the upper Yangtze, the Pearl River and the Red River basins in China. Interestingly, the species ‘*Botia’ zebra* from the Pearl River basin combines a number of morphological characters that otherwise are found in the diploid genus *Leptobotia* with morphological characters of the tetraploid genus *Sinibotia*, therefore the aim of the present study is to test weather ‘*B*.’ *zebra* is the result of a hybridisation event between species from different subfamilies with different ploidy level. A closer morphological examination indeed demonstrates a high similarity of ‘*B*.’ *zebra* to two co-occurring species, the diploid *Leptobotia guilinensis* and the tetraploid *Sinibotia pulchra*. These two species thus could have been the potential parental species in case of a hybrid origin of ‘*B*.’ *zebra*. The morphologic analysis further reveals that ‘*B*.’ *zebra* bears even the diagnostic characters of the genera *Leptobotia* (Leptobotiinae) and *Sinibotia* (Botiinae). In contrast, a comparison of six allozyme loci between ‘*B*.*’ zebra*, *L*. *guilinensis* and *S*. *pulchra* showed only similarities between ‘*B*.*’ zebra* and *S*. *pulchra*, not between ‘*B*.*’ zebra* and *L*. *guilinensis*. Six specimens of ‘*B*.’ *zebra* that were cytogenetically analysed were tetraploid with 4n = 100. The composition of the karyotype (18% metacentric, 18% submetacentric, 36% subtelocentric and 28% acrocentric chromosomes) differs from those of *L*. *guilinensis* (12%, 24%, 20% and 44%) and *S*. *pulchra* (20%, 26%, 28% and 26%), and cannot be obtained by any combination of genomes from *L*. *guilinensis* and *S*. *pulchra*. Phylogenetic reconstructions based on sequence data of the mitochondrial cytochrome *b* gene and the nuclear RAG-1 gene invariably places ‘*Botia’ zebra* as sister species to *S*. *pulchra*, while *L*. *guilinensis* is only distantly related. The presented combination of genetic data demonstrates that ‘*B*.’ *zebra* is not the result of a hybridisation, but a species of tetraploid genus *Sinibotia* with a striking morphological evolution towards an enormous similarity with a co-occurring, but not directly related species. The complete lack of knowledge of the ecology of these species, their main predators or their ecological interactions hampers any conclusion regarding the evolutionary advantage of such adaptation.

## Introduction

One of the most efficient barriers for horizontal gene flow between vertebrate animals is a difference in ploidy level [1,2]. While it might not prevent an original hybridisation event and in many cases not the viability of the F1-offspring, it generally terminates the reproduction line of such hybrids by sterility of the offspring [3,4,5]. In some exceptional cases, the resulting hybrids can make it through with clonal and/or asexual reproduction [1], but due to the absence of gene flow and recombination, offspring of such lineages resemble F1 hybrid individuals and such lineages often are not long lasting. This general rule has been observed in plants as well as in animals, and exceptional cases are very rare, especially among animals. Therefore, any evolutionary successful case of a hybridisation between parental species that differ in ploidy level would provide an interesting model to study the limits of polyploidy as barrier for horizontal gene flow.

Freshwater fishes of the family Botiidae (Cobitoidea: Cypriniformes) are widespread across East, Southeast and South Asia [6,7]. Many species are valued as ornamental fishes worldwide and as tasty food fishes in the area of occurrence. The monophyly of the family has been demonstrated by morphological as well as genetic data [7–11]; and phylogenetic reconstructions of the family revealed two major, long-time separated lineages, which are referred to as subfamilies Leptobotiinae and Botiinae [11,12]. The most remarkable difference between the two subfamilies comes from cytogenetics: all studied Leptobotiinae are diploid with a chromosome number of 2n = 50, while all Botiinae are tetraploid with 4n = 98–100 [12,13]. It has been hypothesised [12] that the difference in ploidy level has played an important role in the separation of the two lineages, since it represents an efficient barrier for hybridisation between the lineages. Both subfamilies have a similar number of species, which was used to claim that there is no obvious difference in the evolutionary success of diploid or tetraploid animals [14]. Leptobotiinae occur in the northern half of the total distribution area (north of the Mekong basin—China, Japan, eastern Russia, northern Vietnam), while most Botiinae live in the southern half of the total distribution area (Mekong and areas south and west of Mekong—from Pakistan to Laos, Malay Peninsula, Indonesia) [7]. However, Leptobotiinae and Botiinae co-occur in the upper Yangtze, the Pearl and the Red River basins, where the genus *Sinibotia* (belonging to Botiinae) is distributed with five recognised species in the area that otherwise is inhabited by Leptobotiinae (a sixth species of *Sinibotia* in the upper Mekong lives outside the range of Leptobotiinae) [7,15].

**Fig 1:**
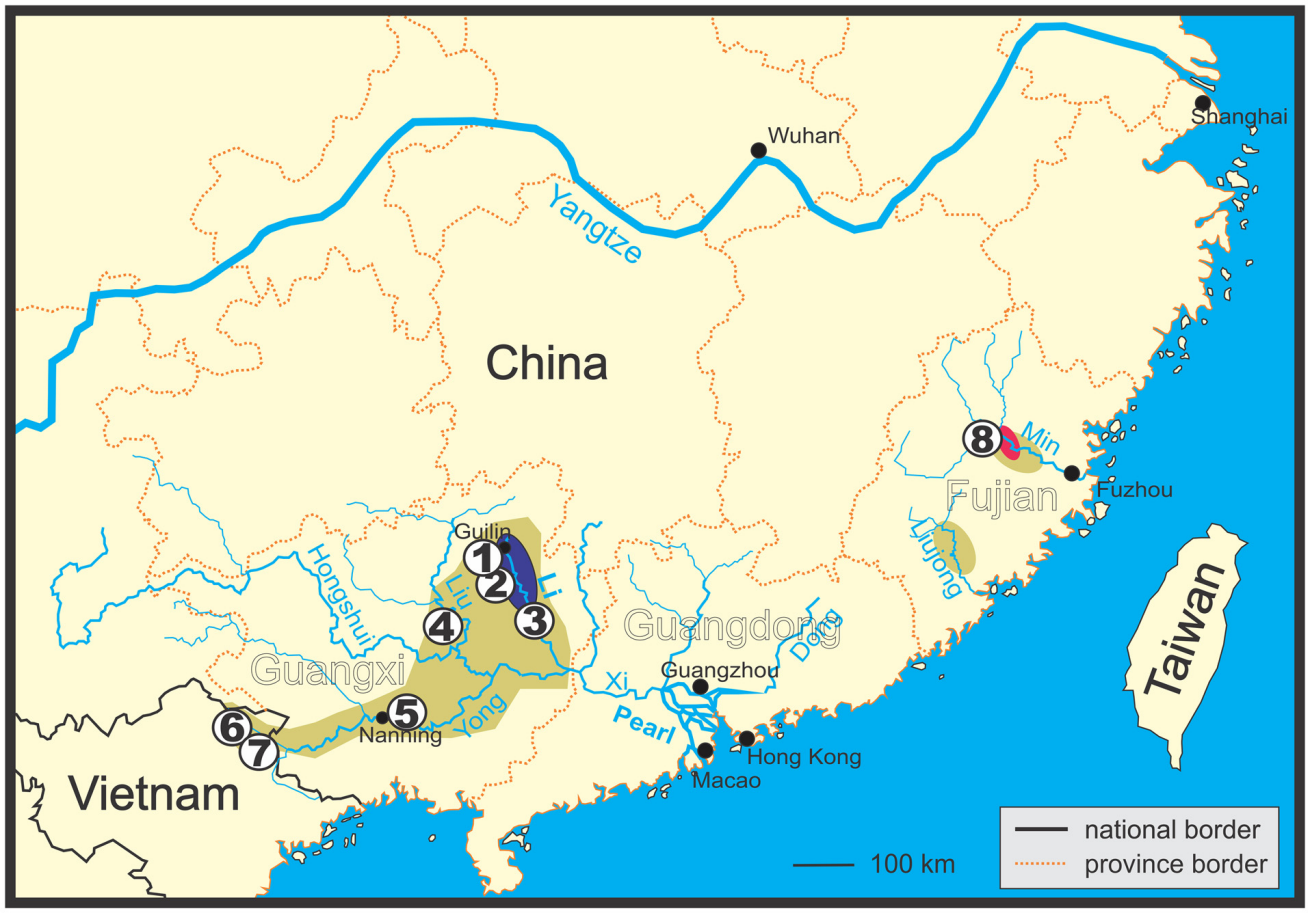
Map of Pearl River basin, Liujong River and Min River in southern China. Distribution areas of botiid species indicated as follows: Green: *S*. *pulchra*, violet: joint distribution area of *L*. *guilinensis* and ‘*B*.’ *zebra*, pink: new record of ‘*B*.’ *zebra*. Circles with numbers indicate geographical origin of analysed samples. Numbers correspond to locality numbers in Table 1.

At least seven species of the genera *Leptobotia*, *Parabotia* and *Sinibotia* occur in the River Li, a northern tributary of the River Xi, Pearl River basin, in southern China [16,17], with two of them being endemic to this river: *Leptobotia guilinensis* Chen, 1980 and ‘*Botia*’ *zebra* Wu, 1939. Since the latter is bearing the diagnostic character of the genus *Leptobotia*, a simple suborbital spine (versus bifid in all other genera of Botiidae), and generally shows a close similarity to the sympatric *Leptobotia guilinensis*, ‘*Botia*’ *zebra* was placed into *Leptobotia* [16]. However, in a phylogenetic analysis basing on the mitochondrial cytochrome *b* gene, ‘*B*’. *zebra* was found to be more closely related to the genus *Sinibotia*, especially to a species that occurs in the River Li, *S*. *pulchra* [18]. One of the possible explanations for a strong discrepancy between morphological and mitochondrial characters, respectively, is mitochondrial introgression, a process where an initial hybridisation is followed by repeated back-crossing events with the paternal species; leading to a morphology that is closer to the paternal species, but a mitochondrial genome that is close to the maternal species. In ‘*Botia*’ *zebra* the morphology is similar to *Leptobotia guilinensis*, but the mitochondrial genome close to *Sinibotia pulchra* and all three species co-occur in the upper River Li (Fig 1). We therefore hypothesise that the evolutionary history of ‘*Botia*’ *zebra* included a hybridisation event between *L*. *guilinensis* and *S*. *pulchra* and test this hypothesis in the present study. Such a hybridisation event between a diploid and a tetraploid species would refute the general assumption that differences in ploidy level represent an efficient barrier against hybridisation and would be of general interest for evolutionary biology.

In the present study, we compare *L*. *guilinensis*, *S*. *pulchra* and ‘*Botia*’ *zebra* using morphologic, cytogenetic, allozyme variability as well as mitochondrial and nuclear DNA sequence characters to test if the later reveals any trace of a past hybridisation between the first two species.

## Material & Methods

### Specimens

Live individuals of *L*. *guilinensis* (eight individuals), *S*. *pulchra* (two individuals) and *S*. *zebra* (six individuals) were obtained together in one mixed group from an import for the ornamental fish trade (Aquarium Glaser, 63110 Rodgau, Germany). Live fishes were kept in the fish housing facilities in Institute of Animal Physiology and Genetics, 277 21 Liběchov, Czech Republic in 60 l tanks with flow-through water of 22°C at a light;dark cycle of 10:14 h. Fishes were fed ad libitum with life *Tubifex* worms. Ethanol or formalin fixed specimens were obtained from local food markets in Guilin (25°16’N, 110°17’E), Mengshan (24°12’N, 110°31’E), Fuzhou (26°04’N, 119°18’E) and Nanning (22°48’N, 108°21’E) in the provinces Guangxi and Fuxien in southern China. A total number of 108 individuals of the three species from 9 localities across the whole distribution area of the species has been analysed (Table 1). Additional 33 specimens of 26 other species as comparative material were obtained from the ornamental fish trade (AquaGlobal, 16356 Werneuchen, Germany). Vouchers are deposited in the collection of the Laboratory of Fish Genetics, IAPG AS CR, Liběchov. All experimental procedures involving fishes during this study were approved by the Institutional Animal Care and Use Committee of the Institute of Animal Physiology and Genetics of the Academy of Sciences of the Czech Republic, according with directives from the State Veterinary Administration of the Czech Republic, permit number 155/2012, and by permit number CZ 02386 from the Ministry of Agriculture of the Czech Republic.

**Table 1:** Number of specimens and geographical origin of *Leptobotia guilinensis*, ‘*B*.’ *zebra* and *Sinibotia pulchra* analysed in the present study.

Locality number	Species	n	Sample ID	Locality	River	Drainage	Country	Province
1	L. guilinensis	23	A8861-8883	market in	upper Li	Pearl	China	Guangxi
	L. guilinensis	3	A8901-8903	Guilin	River	River		
	L. guilinensis	2	A1798-1799		(漓江)	(珠江)		
	B. zebra	2	A8905-8906					
	S. pulchra	3	A8889-8891					
	S. pulchra	1	A8900					
	S. pulchra	6	A1782-1787					
2	L. guilinensis	10	A5267-5276	ornamental	upper Li	Pearl	China	Guangxi
	B. zebra	6	A5277-5282	fish import	River (漓江)	River		
	S. pulchra	2	A5286-5287			(珠江)		
3	B. zebra	5	A8604-8608	market in Mengshan	upper Li River (漓江)	Pearl River (珠江)	China	Guangxi
4	S. pulchra	1	A8993	Liu River above Yizhou	middle Liu River (柳江)	Pearl River (珠江)	China	Guangxi
5	S. pulchra	5	A9102-9106	market in Nanning	Yong River (邕江)	Pearl River (珠江)	China	Guangxi
6	S. pulchra	1	A8395	Bang Giang at Cao Bang city	Bang Giang (*Sông Bằng)*	Pearl River (珠江)	Vietnam	Cao Bang
7	S. pulchra	4	A8397-8400	stream in Hoa An district	Bang Giang (*Sông Bằng)*	Pearl River (珠江)	Vietnam	Cao Bang
8	B. zebra	1	A8614	unknown	Min River	Min River	China	Fujian
	S. pulchra	24	A8615-8638		(閩江)	(閩江)		
9	L. guilinensis	3	A0205-0208	ornamental	Details	unknown		
	S. pulchra	2	A3681-3682	fish import				
	S. pulchra	4	A0015-0018					

All listed specimens were used in the morphological analyses, but not all for the other methods. Locality number matches the numbers given in Fig 1; n = number of specimens; sample ID is the individual number given to each specimen (collection numbers IAPG).

### Morphology

Morphological measurements were taken from 26 specimens of *L*. *guilinensis*, *S*. *pulchra* and *S*. *zebra* with digital callipers point-to-point according [19]. Important morphologic characters and pigmentation were estimated from nearly all specimens either directly in the case of fixed specimens or from photos taken of live specimens. Preparations of suborbital spines of nine specimens were carried out under an Olympus SZX7 stereomicroscope equipped with a u-Eye camera.

### Chromosome analysis

Mitotic chromosomes were obtained from regenerated fin tissue as described by [20,21] with slight modifications. Briefly, the posterior margin of the caudal fin was cut off and three weeks later, the regenerated tissue of the fin was collected to be incubated in Ringer solution with 0,025% colchicine for about two hours at room temperature. Cells were fixed in a mixture of methanol and acetic acid (3:1) at 4°C for 25 min. This step was repeated three times. The fixed tissue was minced in 50% acetic acid and drops of the resulting suspension were placed on pre-heated slides (50°C) and sucked back after 20 sec. The slide was dried at room temperature and stained for 10 min in 5% Giemsa solution (pH 6.8) (Merck, Darmstadt, Germany) before examination of metaphase plates with an Olympus AX70 light microscope. From 17 live individuals that were available for the analyses, results with satisfying quality were obtained from nine individuals (five *L*. *guilinensis*, two *S*. *pulchra*, two *‘B*.*’ zebra*). The number of chromosomes of at least 20 metaphase plates per individual was counted. Chromosome morphology was classified as m—metacentric, sm—submetacentric, st—subtelocentric, a—acrocentric [22].

### Allozyme analysis

Fin tissue was homogenised with an equal amount of buffer (0.1 mol/l Tris-HCl pH 8.5) and centrifuged for clarifying. All manipulations with tissue were carried out on ice. Electrophoresis on starch gel was carried out in a refrigerator. Six allozyme loci (glucosephosphate isomerase Gpi-A, aspartate amino transferase s-Aat, malate dehydrogenase s-Mdh A, lactate dehydrogenase Ldh A and Ldh B, phosphoglucomutase Pgm) were stained [23,24]. Altogether 18 individuals were studied. Loci Gpi-A and Pgm were analysed in three and two buffer systems, respectively (F [25], MC2 [26], V [27]).

### DNA sequence analyses

Genomic DNA was isolated from fin tissue samples using the DNeasy Tissue kit (Qiagen, Hilden, Germany) according to manufacturer’s instructions. The mitochondrial cytochrome *b* (cyt *b*) was amplified and sequenced using the primers Glu-L.Ca14337-14359: 5’- GAA GAA CCA CCG TTG TTA TTC AA– 3’ and Thr-H.Ca15568-15548: 5’- ACC TCC RAT CTY CGG ATT ACA– 3’ [12]. An approximately 970 bp long portion of RAG-1 was amplified using the primers RAG-1F (5’-AGCTGTAGTCAGTAYCACAARATG-3’ [28]) and RAG-RV1 (TCCTGRAAGATYTTGTAGAA-3’ [10]) or RAG-8R (5’-CGC CAC ACA GGY TTC ATC T-3’ [28]. Same primers were used also for sequencing reactions. PCR amplifications were performed in 25 μl reaction volumes of 10 mM Tris-HCl, 50 mM (NH_4_)_2_SO_4_, 0.1% of Triton X-100, 1.5 mM MgCl_2_, 2 mM TMA oxalate (PCR enhancer), containing 5 nmol of each nucleotide, 1.25 U of *Taq* polymerase (all chemicals Top-Bio, Prague, Czech Republic) and 12.5 pmol of each primer.

The PCR reaction profile (MJ Research thermocycler) included 5 min of initial denaturation at 95°C, touch-down profile of 1 min at 94°C, 1 min 30 s at 60–55°C (1°C/cycle) and 2 min at 72°C, followed by 30 cycles with annealing temperature held at 54°C. The reaction was completed by final extension at 72°C for 7 min.

PCR products were purified by QIAquick PCR Purification Kit (Qiagen). Forward and reverse sequencing reactions were performed with BigDye^™^ Terminator Cycle Sequencing Kit v.1.1 (PE Applied Biosystems, Darmstadt, Germany) according to manufacturer’s instructions and products purified with DyeEx Spin Kit (Qiagen). Sequencing was performed on ABI Prism 3130 (Applied Biosystems).

Chromatograms were assembled using SeqMan Pro 10.1.2 of the LaseGene software package (DNASTAR). The sequences were aligned and manually revised in BioEdit 7.0.5.3 [29] and evaluated based on their amino acid translation.

The newly obtained data were deposited in GenBank under the accession numbers KU517025-KU517132.

We have analysed molecular data from altogether 102 individuals of Botiidae representing 29 species currently considered as valid. Based on the former studies on Cobitoidea [10,30], we have selected *Gyrinocheilus aymonieri* as outgroup.

The molecular datasets were analysed using the Bayesian inference in MrBayes 3.2.2 [31]. The two genes were analysed separately with the aim to see potential discrepancies between mitochondrial and nuclear markers. The datasets were partitioned into codon positions. Prior to the analyses, the MEGA 5.10 software [32] was used to estimate the most suited model for each gene partition under the Bayesian information criterion (BIC). The Bayesian analyses were performed in two independent runs of 5 million generations, each using six Markov Chains, starting with random trees and sampling frequency set to 100 generations. The parameter settings corresponded to the best-fit models. The log-likelihood score distribution was examined in order to assess if stationarity was reached. The first 5000 saved trees were discarded as the burn-in and a 50% majority rule consensus of the remaining trees was computed. Statistical support of clades was assessed by posterior probabilities.

## Results

### Morphology

#### Morphometry

- In 13 out of 33 morphometric and meristic characters there was no overlap of measurements between *L*. *guilinensis* and *S*. *pulchra* (Category A, Table 2), while in further 12 characters, the overlap was small (Category B). In the remaining eight characters the overlap was large (Category C); therefore these characters were unsuited to evaluate a morphological similarity between *‘B*.*’ zebra* and the two potential parental species. However, in one of these ‘uninformative’ characters in Category C (Number of branched dorsal-fin rays), seven out of eight specimens of ‘*B*.’ *zebra* showed a character state that was observed in neither *L*. *guilinensis* nor *S*. *pulchra*, indicating an autapomorphy of *‘B*.*’ zebra*. When comparing *‘B*.*’ zebra* with *L*. *guilinensis* and *S*. *pulchra*, it shared the range of measurements with *S*. *pulchra* in two characters of Category A and in two characters of Category B, with *L*. *guilinensis* in six characters of Category A and in eight characters of Category B, while its range was intermediate between the two species in five characters of Category A and in two characters of Category B.

**Table 2:** Morphometric comparison of *Leptobotia guilinensis*, *Sinibotia pulchra* and ‘*Botia*’ *zebra*.

	*Leptobotia guilinensis*	‘*Botia*’ *zebra*	*Siibotia pulchra*	Comparison
	n = 10	n = 8	n = 10	
**A. Characters without overlap between *Leptobotia guilinensis* and *Sinibotia pulchra***				
Pre-pelvic length	51–54	56–59	55–59	zebra = pulchra
Preanal length	74–77	78–80	78–80	zebra = pulchra
Dorsal head length	16–20	20–22	21–24	zebra intermediate
Snout length	6–8	10–11	12–14	zebra intermediate
Pre-anus length	63–71	70–73	73–76	zebra intermediate
Lateral head length	21–24	24–26	27–30	zebra intermediate
Head depth at eye	8–9	10–11	11–12	zebra intermediate
Head depth at nape	11–13	12–14	15–16	zebra = guilinensis
Maximum body depth	11–16	13–17	19–23	zebra = guilinensis
Body depth at dorsal origin	11–17	13–17	18–23	zebra = guilinensis
Maximum head width	8–10	8–10	11–13	zebra = guilinensis
Head width at nares	4–5	4–6	6–9	zebra = guilinensis
Body width at anal origin	4–7	5–6	8–10	zebra = guilinensis
**B. Characters with slight overlap between *Leptobotia guilinensis* and *Sinibotia pulchra***				
Predorsal length	49–58	55–60	55–62	zebra = pulchra
Number of pectoral-fin rays	11–13	13–15	13–15	zebra = pulchra
Interorbital width	3–4	4–4	4–6	zebra intermediate
Length of caudal peduncle	14–18	13–16	12–14	zebra intermediate
Length of upper caudal lobe	16–21	18–20	20–25	zebra = guilinensis
Length of pectoral fin	11–14	12–14	14–19	zebra = guilinensis
Length of lower caudal lobe	18–21	18–21	20–26	zebra = guilinensis
Body width at dorsal origin	6–10	6–9	10–15	zebra = guilinensis
Depth of caudal peduncle	10–13	10–13	13–14	zebra = guilinensis
Length of pelvic fin	10–12	10–11	12–15	zebra = guilinensis
Length median caudal rays	7–10	7–9	9–15	zebra = guilinensis
Total length	116–121	116–120	119–127	zebra = guilinensis
**C. Characters with broad overlap between *Leptobotia guilinensis* and *Sinibotia pulchra***				
Eye diameter	2–3	2–3	3–3	
Depth of anal fin	13–15	12–15	14–17	
Postorbital length	12–14	13–14	13–15	
Height of dorsal fin	11–16	11–14	12–18	
Branched dorsal-fin rays	8 ½	7(8) ½	8 ½	zebra speciality
Branched caudal-fin rays	9+8	9+8	9+8	
Branched anal-fin rays	5	5	5	
Number of pelvic-fin rays	8	8	8	

Values give range as % of standard length. Under ‘Comparison’ is indicated if ‘*Botia*’ *zebra* has values like one of the potential parental species or if it is intermediate.

#### Pigmentation pattern

**Fig 2:**
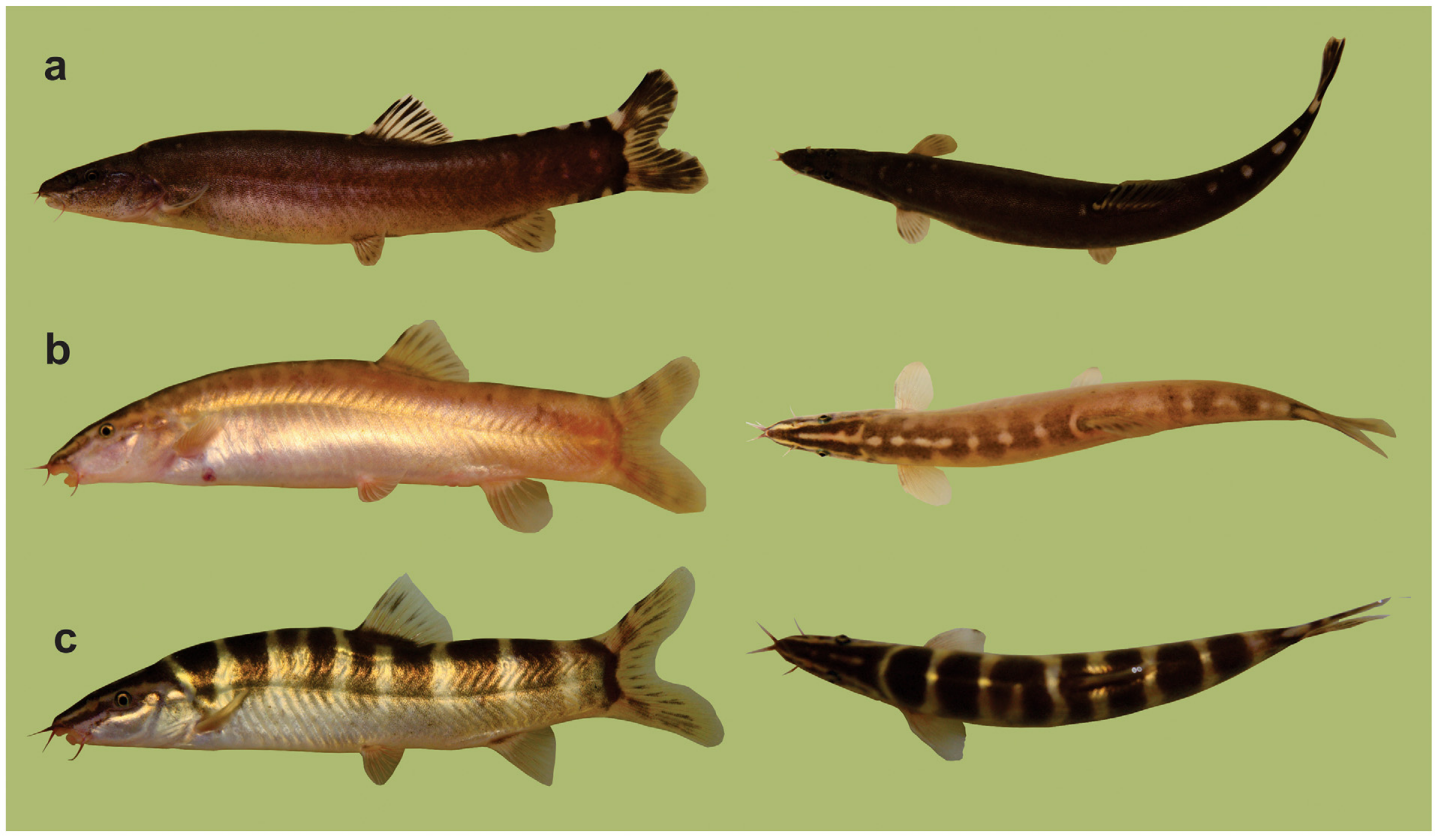
Habitus of *Leptobotia guilinensis*, *Sinibotia pulchra* and *’B*.*’ zebra*. Specimens of (a) *Leptobotia guilinensis*, (b) ‘*Botia*’ *zebra* and (c) *Sinibotia pulchra* in lateral and dorsal view. ‘*Botia*’ *zebra* shares the general body shape and pigmentation pattern, the smaller head size and the shorter, round fins with *L*. *guilinensis*, but the pigmentation of the head with *Sinibotia pulchra*.

- The pigmentation pattern of *S*. *pulchra* is much like that of all other members of the genus *Sinibotia*: Broad dark brown bars run from one body side across the back to the other side, reaching nearly always below lateral midline and regularly to level of pelvic fin origin (Fig 2). In small and medium sized individuals 6–10 bars are present, much broader than interspaces, but in larger individuals each bar might split into two. On the dorsal side of the head run two dark stripes from the snout to the neck and one on each side of the head from the snout through the eye. Between the dark stripes are two prominent light stripes, a long one from the snout to the end of the operculum and a short along the dorsal midline of the head. In *Leptobotia guilinensis*¸ body and head are homogenously light to dark brow with a lighter belly. Prominent light blotches are present along the dorsal midline, but often only visible behind the base of the dorsal fin. Dark saddles are sometimes visible between the light blotches, but usually too faint to figure out the precise number and outline. A thin black stripe runs from the snout to the eye, but no light stripes are present. In ‘*B*.’ *zebra* the body sides are uniformly brown like in *L*. *guilinensis*, but usually in lighter brown. On the back, faint saddles are sometimes visible, often hard to see, never reaching to lateral midline. In some specimens the saddles are present only in the anterior part of the body, but if present along whole back their number is higher than ten. Between the saddles are light blotches, very similar to the light blotches in *L*. *guilinensis*, often merging into a line in the anteriormost part of the dorsum. On the head are dark stripes from the snout to the neck and from the snout through the eye two and prominent light stripes between them as described for *S*. *pulchra*. In general, ‘*B*.’ *zebra* combines the head pigmentation of *S*. *pulchra* with the body pigmentation of *L*. *guilinensis*.

#### Suborbital spine

**Fig 3:**
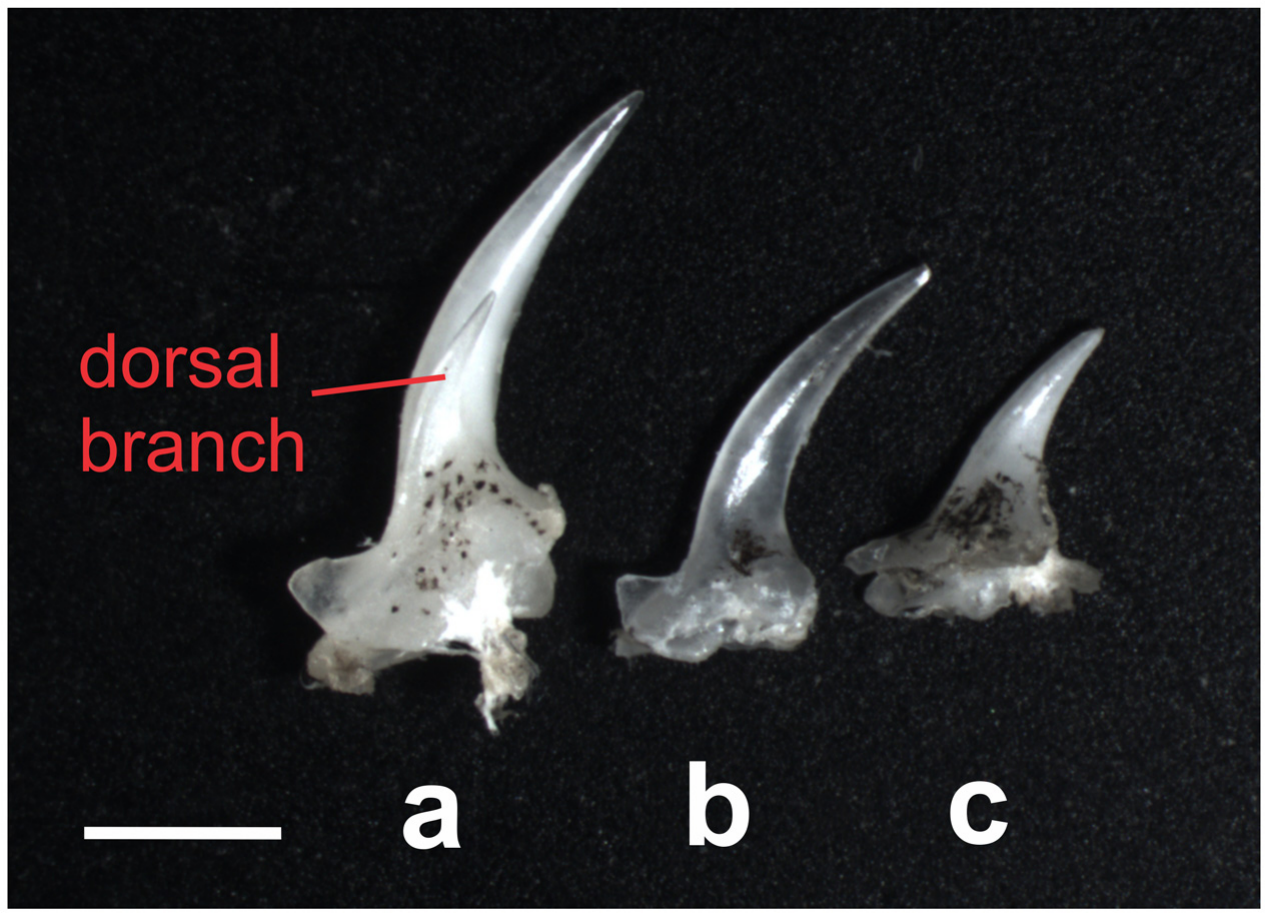
Suborbital spines of *Leptobotia guilinensis*, *Sinibotia pulchra* and *’B*.*’ zebra*. Right suborbital spine of (a) *Sinibotia pulchra* (A1783, 70.5 mm SL), (b) ‘*Botia*’ *zebra* (A8607, 61.9 mm SL) and (c) *Leptobotia guilinensis* (A8573, 70.6 mm SL) in dorsal view. The spine bears a dorsal branch in *S*. *pulchra*, but this branch is missing in *L*. *guilinensis* and ‘*B*.’ *zebra*. A simple (= unbranched) suborbital spine is the diagnostic character for the genus *Leptobotia*. Scale bar is 1 mm.

–The suborbital spine is an erectable spine formed by the lateral ethmoid bone and located in a skin pocket below each eye. It is present in all members of the family Botiidae as well as in the distantly related families Cobitidae and Serpenticobitidae and its shape is of taxonomical value. In all species of *Leptobotia*, including *L*. *guilinensis*, this spine is simple, meaning it has a single branch and tip [7]. In all other Botiidae, including *S*. *pulchra*, the spine is double, meaning it has a main and a side branch and two tips [7]. In ‘*B*.’ *zebra*, the spine turned out also to be simple, like in *Leptobotia* (Fig 3).

#### Mental lobes

**Fig 4:**
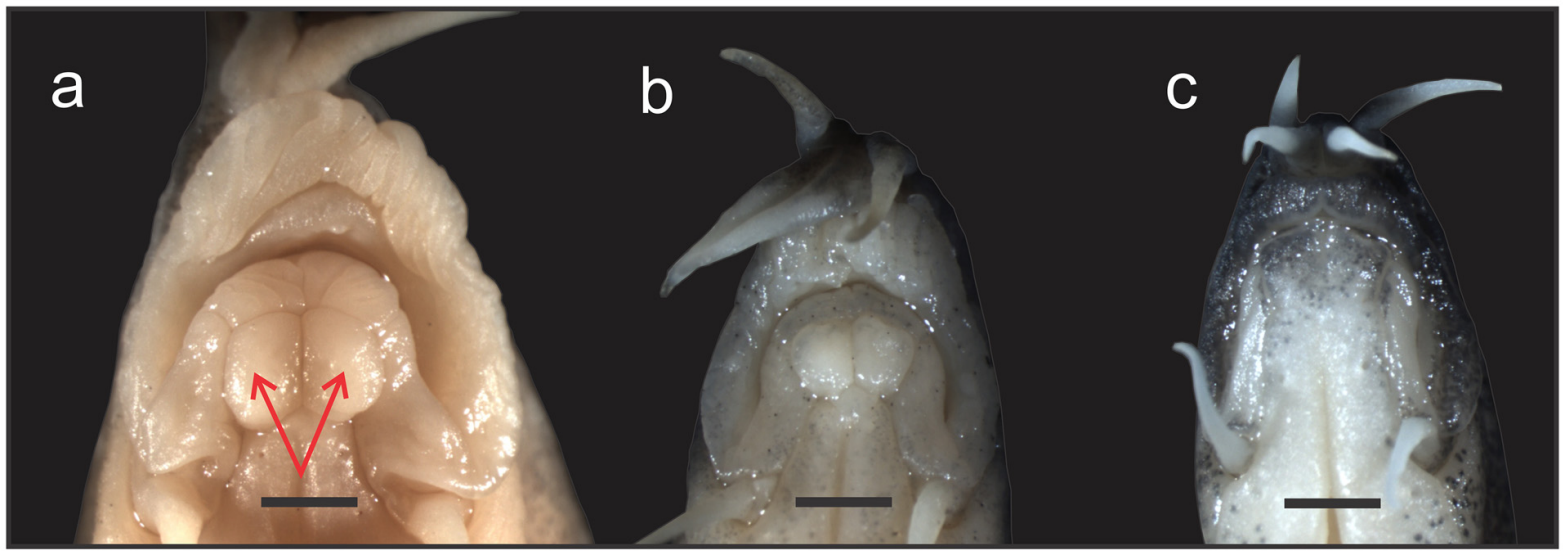
Mouthes of *Leptobotia guilinensis*, *Sinibotia pulchra* and *’B*.*’ zebra*. Mouth of a) *Sinibotia pulchra*, A 9102, 73.0 mm SL; b) ‘*B*.’ *zebra*, A 8904, 63.1 mm SL and c) *Leptobotia guilinensis*, A8868, 71.6 mm SL in ventral view. The presence of two prominent buttons on the lower lip (arrows in *S*. *pulchra*) are a diagnostic character of the genus *Sinibotia*. Scale bar is 1 mm.

–In many species of Botiidae the lower lip develops two median extensions, called mental lobes, and presence and shape of these extensions are important taxonomic characters [7]. In all species of *Sinibotia* the extensions are present, large and of oval or kidney-like shape [7,15]. This shape of the mental lobes is considered a diagnostic character for the genus *Sinibotia* [7]. In all analysed specimens of *S*. *pulchra* the mental lobes were present, large and had the shape characteristic for *Sinibotia*, while in all analysed specimens of *L*. *guilinensis* no mental lobes were present. In all analysed specimens of ‘*B*.’ *zebra* mental lobes were present, large and had the shape characteristic for *Sinibotia* (Fig 4).

The literature names additional characters to distinguish between *Leptobotia* and *Sinibotia*, namely the presence of scales on the cheeks and of a pario-frontal fontanelle in *Leptobotia* (vs. both characters absent in *Sinibotia*) [7,33,34]. Since both turned out to be absent in five dissected specimens of *L*. *guilinensis*, these characters are not truly diagnostic and were unsuited for the comparison in the given case.

### Chromosome analysis

**Fig 5:**
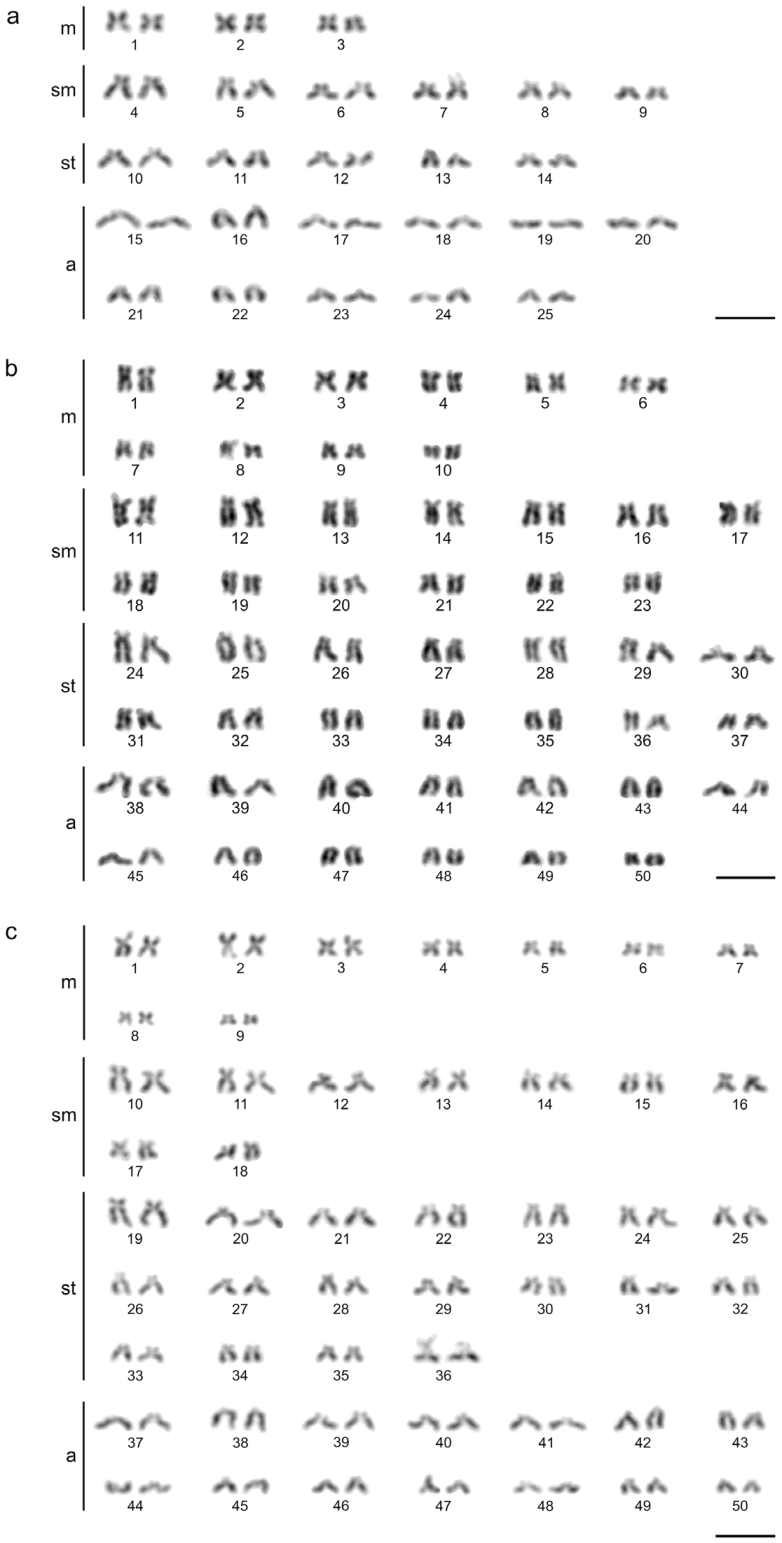
Karyotypes of *Leptobotia guilinensis*, *Sinibotia pulchra* and *’B*.*’ zebra*. Karyotypes of the diploid species *Leptobotia guilinensis* (a) and the tetraploid species ‘*Botia*’ *zebra* (b) and *Sinibotia pulchra* (c). Bar = 10 μm.

Metaphases of suited quality for further analyses were obtained from five individuals of *L*. *guilinensis*, two ‘*B*’ *zebra* and two *S*. *pulchra*. The diploid chromosome number of all analysed *L*. *guilinensis* was 2n = 50, proving the diploid status of these individuals, while all individuals of ‘*B*’ *zebra* and *S*. *pulchra* were tetraploid with a chromosome number of 4n = 100 (Table 3, Fig 5). Karyotypes of all analysed species were composed of comparatively small chromosomes, slightly decreasing in size. Especially in the tetraploid species chromosomes were generally very small, with their centromere positions gradually ranging from median to nearly terminal making the borderlines between formal chromosomal categories questionable in a small subset of chromosomal pairs.

**Table 3:** Chromosme numbers and karyotype composition of *Leptobotia guilinensis*, *Sinibotia pulchra* and *’B*.*’ zebra*.

	n	m	sm	st	a
*Leptobotia guilinensis*	50	6	12	10	22
*Sinibotia pulchra*	100	20	26	28	26
*‘Botia’ zebra*	100	18	18	36	28

Chromosomal characteristics of *Sinibotia pulchra*, ‘*Botia*’ *zebra* and *Leptobotia guilinensis* from the upper Li River (Pearl River basin) including diploid chromosome number (2n) and karyotype description.

### Allozyme analysis

In three (s-Aat, Ldh A, Ldh B) of the six analysed loci alleles were shared between the three analysed taxa and therefore were not informative for the given study. In all informative loci (Gpi-A, s-Mdh A, Pgm), *S*. *pulchra* shared alleles with *‘B’*. *zebra*, but both did not share alleles with *L*. *guilinensis* (Table 4). Therefore the allozyme data suggest a high similarity between *S*. *pulchra* and *‘B*.*” zebra*, while *L*. *guilinensis* appears to be more distantly related.

**Table 4:** Allozymes of *Leptobotia guilinensis*, *Sinibotia pulchra* and *’B*.*’ zebra*.

Species	n	Gpi-A	Gpi A	Gpi A	s-Aat	s-Mdh A	Ldh-A	Ldh-B	Pgm	Pgm
Buffer		V	MC 2	F	MC 2	MC 2	V	V	V	F
*Sinibotia pulchra*	4	055	037	039	096	A, C	030, 136	053, 067	083	080
*‘Botia’ zebra*	6	055	037	039	096	A	030	053, 067	083	080
*Leptobotia guilinensis*	8	065, 070,083, 099	050, 064,078, 095	060, 078, 096	084, 096	B	136	053, 067	107	100

Presence of six allozymes in *Sinibotia pulchra*, ‘*Botia*’ *zebra* and *Leptobotia guilinensis*.

### DNA sequence analysis

Table 5 summarises the species and individuals analysed in this study including the novel sequences generated as well as those that were obtained from GenBank.

**Table 5:** Specimens of Botiidae used in the DNA analyses.

*Species*	ID	Cyt b	RAG
*Ambastaia nigrolineata*	A0031	AY887845	EF056329
*Ambastaia sidthimunki*	A0183	AY887842	KU517025
	KP319024	KP319024	
*Botia dario*	A7553	KU517084	KU517026
	EU409614		EU409614
*Botia histrionica*	A0041	AY887794	KU517027
*Botia lohachata*	A0426	KU517085	KU517028
*Botia striata*	A0011	AY887783	KU517029
	EU711109		EU711109
*Chromobotia macracanthus*	A0178	AY887840	KU517030
	A0179	AY887841	KU517031
	EU711137		EU711137
	JN177192		JN177192
*Leptobotia elongata*	A0214	AY887779	KU517032
	A8392	KU517086	KU517033
	JN177196	-----	JN177196
*Leptobotia guilinensis*	A0124	AY887780	KU517034
	A0205	AY887781	KU517035
	A1798	KU517087	KU517036
	A1799	KU517088	KU517037
	A5267a, k	KU517089	KU517038
	A5268 a	KU517090	KU517039
	A5269 a, k	KU517091	KU517040
	A5270 a, k	KU517092	KU517041
	A5271 a	KU517093	KU517042
	A5273 a, k	KU517094	KU517043
	A5277 a, k	KU517095	KU517044
	A5279 a	KU517096	KU517045
	A8569	KU517097	KU517046
	A8570	KU517098	KU517047
*Leptobotia microphthalma*	A5283	KU517099	KU517048
	A5285	KU517100	KU517049
*Leptobotia pellegrini*	A1459	KU517101	KU517050
	A1813	KU517102	KU517051
	EU292683	-----	EU292683
*Leptobotia taeniops*	A8544	KU517103	KU517052
	A8545	KU517104	KU517053
	JN177193	-----	JN177193
	JN177194	-----	JN177194
*Parabotia banarescui*	A0217	AY887782	KU517054
*Parabotia bimaculata*	JN177197	-----	JN177197
*Parabotia fasciata*	A8391	KU517105	KU517055
*Parabotia lijiangensis*	JN177199	-----	JN177199
*Parabotia mantschuricus*	EU711138	-----	EU711138
*Sinibotia pulchra*	A0015	AY887800	-----
	A0016	AY887801	-----
	A0121	AY887802	-----
	A0396	AY887803	KU51705
	A0397 a	AY887804	-----
	A1782	KU517106	-----
	A1783	KU517107	KU517057
	A1785	KU517109	KU517058
	A1786	KU517110	KU517059
	A1787	KU517111	KU517060
	A3681 a	-----	-----
	A3682 a, k	-----	-----
	A5287 a, k	KU517112	KU517061
	A8397	KU517113	KU517062
	A8398	KU517114	KU517063
	A8615	KU517115	KU517064
	A8616	KU517116	KU517065
	A0243	KU517117	-----
	AY625705	AY625705	-----
	AY625706	AY625706	-----
	EU282332	EU282332	-----
*Sinibotia robusta*	A0024	AY887805	EF056333
	A2226	KU517118	KU517066
	A2227	KU517119	KU517067
	A2228	-----	KU517068
	A8582	KU517120	KU517069
	A8583	KU517121	KU517070
	A0242	KU517122	-----
	JN177191	-----	JN177191
	AY625707	AY625707	-----
	AY625708	AY625708	-----
	DQ105208	DQ105208	-----
*Sinibotia supercillaris*	JN177190	-----	JN177190
	AY625702	AY625702	-----
	AY625703	AY625703	-----
	AY625704	AY625704	-----
*Sinibotia zebra*	A5272 a, k	KU517123	KU517071
	A5274 a	KU517124	-----
	A5275 a, k	KU517125	KU517072
	A5276 a	KU517126	KU517073
	A5278 a	KU517127	KU517074
	A5280 a	KU517128	KU517075
	A8614	KU517129	KU517076
	DQ105206	DQ105206	-----
	DQ105207	DQ105207	-----
	EU282333	EU282333	-----
	EU282334	EU282334	-----
	EU282335	EU282335	-----
*Syncrossus beauforti*	A0059	AY887816	KU517077
	FJ650411	-----	FJ650411
*Syncrossus berdmorei*	A0277	AY887823	KU517078
*Syncrossus helodes*	A0574	KU517130	KU517079
	GQ174422	-----	GQ174422
*Yasuhikotakia eos*	A0062	AY887829	KU517080
*Yasuhikotakia lecontei*	A0568	KU517131	KU517081
*Yasuhikotakia modesta*	A0200	AY887833	KU517082
	GQ174361	GQ174361	-----
	GQ174419	-----	GQ174419
	JQ346129	-----	JQ346129
*Yasuhikotakia morleti*	A0067	AY887835	KU517083
	GQ174375	GQ174375	-----
	FJ650412	-----	FJ650412
*Gyrinocheilus aymonieri*	A0256	KU517132	EF056390

Species, number code, and Genbank accession numbers of Botiidae used in the DNA sequence analyses, cytogenetic analysis and allozyme analysis. Individuals with GenBank assession number have been included in the DNA sequence analyses, those marked ^a^ were used in allozyme analyses and those marked with ^k^ were karyotyped.

Altogether we have analysed 102 specimens of Botiidae including 49 and 59 novel sequences of cyt *b* (1121 bp) and RAG1 (971 bp), respectively. Into the cytochrome *b* dataset, 14 sequences of *L*. *guilinensis*, 19 sequences of *S*. *pulchra* and 12 sequences of ‘*B*.’ *zebra* were included; in the RAG dataset it were 14, 10 and six sequences, respectively.

The models selected for each partition (codon position) based on BIC score were following: TN93+G+I, HKY+G and GTR+G for the 1st, 2nd and 3rd codon positions of cyt *b*, respectively, and HKY+G, JC+I and JC for the 1st, 2nd and 3rd codon positions of RAG 1, respectively. Those were taken into account for the subsequent Bayesian analyses.

**Fig 6:**
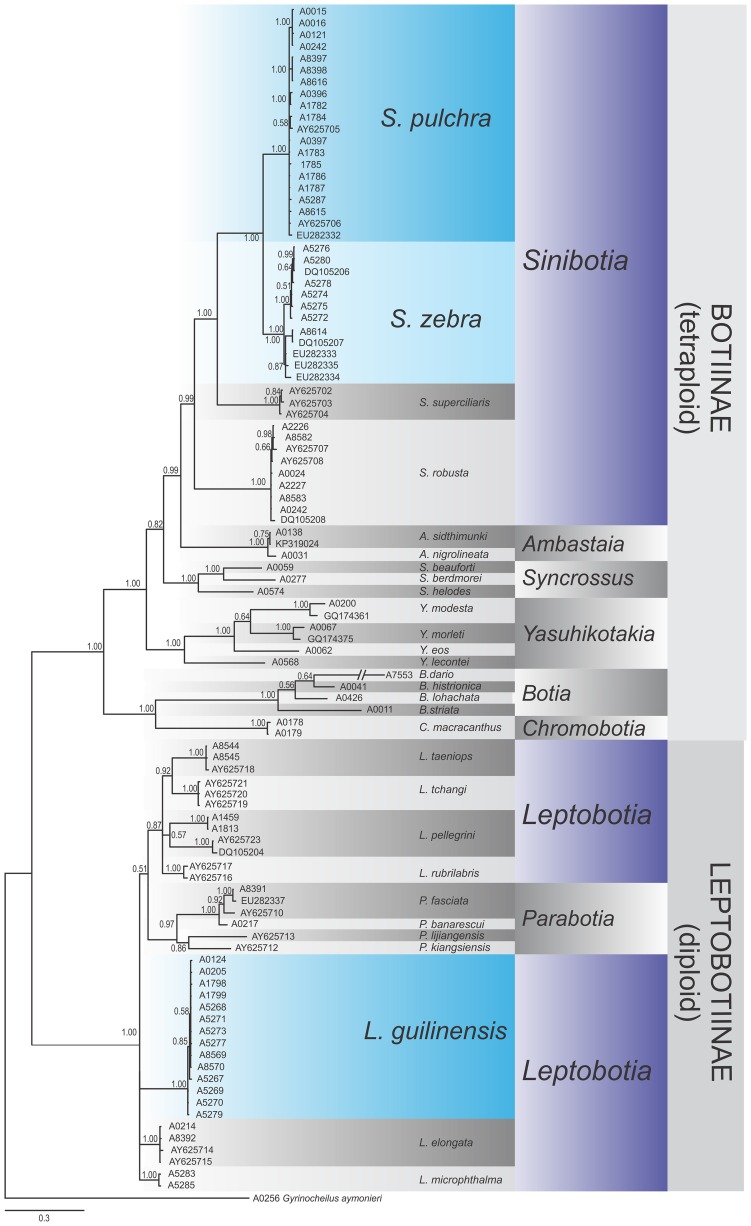
Phylogenetic relationships of Botiidae as revealed by the Cytochrome b dataset. Phylogenetic relationships among freshwater fishes of the family Botiidae as revealed by a Bayesian analyses of the mitochondrial cytochrome *b* gene. The values at the nodes represent the Bayesian posterior probabilities. *Sinibotia zebra* and *Sinibotia pulchra* are sister species, while *Leptobotia guilinensis* is only distantly related.

**Fig 7:**
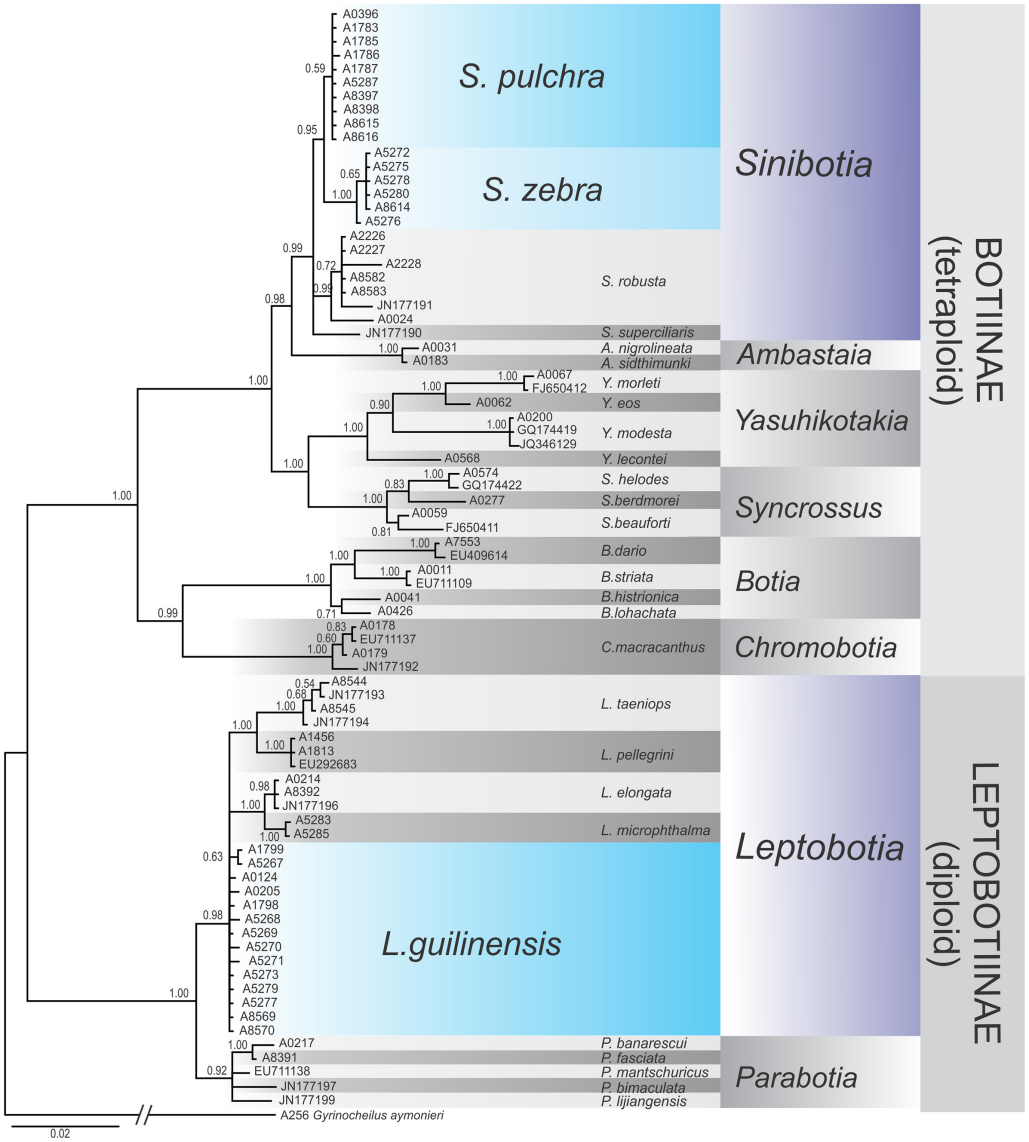
Phylogenetic relationships of Botiidae as revealed by the RAG-1 dataset. Phylogenetic relationships among freshwater fishes of the family Botiidae as revealed by a Bayesian analyses of the nuclear RAG-1 gene. The values at the nodes represent the Bayesian posterior probabilities. *Sinibotia zebra* and *Sinibotia pulchra* are sister species, while *Leptobotia guilinensis* is only distantly related.

Phylogenetic reconstructions of both analysed genes provide generally congruent genealogies: the major split within Botiidae separated the diploid subfamily Leptobotiinae from the tetraploid subfamily Botiinae (Figs 6 and 7). Both datasets identified all described genera as monophyletic lineages with high statistic support except *Leptobotia* in the RAG dataset. These observations are well in agreement with former observations [11,12]. In general, the slower evolving RAG gene brought a better resolution at the older genealogic events, while the faster evolving cytochrome *b* gene had a better resolution around the tips of the trees, which is a well-know characteristics of these two genes. No discrepancies that would indicate a potential hybridisation event were detected.

All specimens of *‘B*.*´zebra*, *L*. *guilinensis* and *S*. *pulchra* form own monophyletic groups, confirming that the three species are unambiguously identifiable by these markers. The lineages of *‘B*.*´zebra* and *S*. *pulchra* show a sister relationship and together are embedded into the comparative material of *Sinibotia*, while the lineage of *L*. *guilinensis* is closely related to all comparative samples of *Leptobotia*, but only distantly related to the lineages formed by *B*.*´zebra* and *S*. *pulchra*.

## Discussion

Our results demonstrate that *‘B*.*´zebra* shows a high morphological similarity to *L*. *guilinensis*, but also shares characters with *S*. *pulchra* and in some characters presents an intermediate morphotype. Moreover, except having 7½ -8½ branched dorsal-fin rays (versus 8½ in to *L*. *guilinensis* and *S*. *pulchra*) it reveals only characters that are also found in either of the two species. The prevalence of synapomorpies with either one of the two other species or an intermediate character state strongly supports the hypothesis of a hybrid origin of *‘B*.*´zebra*. Most important in this respect are the diagnostic characters: the diagnostic character for the genus *Leptobotia* is the simple suborbital spine. In ‘*B*.’ *zebra*, the spine also is simple; therefore it bears the diagnostic character of the genus *Leptobotia* (Fig 3). Consequently, ‘*B*.’ *zebra* was placed into *Leptobotia* [7,16,35,36]. The diagnostic character of the genus *Sinibotia* is the presence of a pair of mental lobes in a button-like; and ‘*B*.’ zebra bears these buttons, meaning it also carries the diagnostic character of the genus *Sinibotia* (Fig 4). This result offers two potential explanations: either *‘B*.*´zebra* is of hybrid origin or the described characters are not diagnostic. As mentioned above, the pigmentation pattern of *‘B*.*´zebra* includes the head pigmentation of *Sinibotia* and the body pigmentation of *L*. *guilinensis*, further strengthening the assumption of a hybrid origin (Fig 2). Therefore all morphological data suggest that ‘*B*.’ *zebra* is a mixture of these two species; that means the product of a hybridisation. As stated above, the different ploidy level of the diploid *L*. *guilinensis* and the tetraploid *S*. *pulchra* should represent an efficient barrier against any horizontal gene flow between these two lineages.

However, a first hybridisation step would be possible, but potentially formed F1 hybrids would be excluded from further reproduction. In order to test the F1 hybrid status of '*B*.' *zebra*, their ploidy level was investigated. All six analysed individuals of '*B*.' *zebra* were tetraploid with a chromosome number of 4n = 100. Consequently, these individuals were no F1 hybrids, inducing strong doubts against the postulate of the efficiency of ploidy level differences as barrier against gene flow. Moreover, the composition of the karyotype of ‘*B*.’ *zebra* turned out to be very similar to that of *S*. *pulchra*, but did not reveal any trace of introduction of one or two chromosome sets of *L*. *guilinensis* into its karyotype. Due to the high number of uni-armed chromosomes in *L*. *guilinensis* the number of uni-armed chromosomes in ‘*B*.*’ zebra* would have elevated considerably in comparison to *S*. *pulchra*. Nevertheless, the number of uniarmed chromosomes is slightly increased in '*B*.' *zebra* when compared to *S*. *pulchra*. Theoretically, this could be the result of a number of back-crossings of the original hybrid with *S*. *pulchra* that brought the karyotype composition of ‘*B*.*’ zebra* closer to that of *S*. *pulchra*, while some chromosomes of *L*. *guilinensis* are still present, but not distinguishable from *Sinibotia* chromosomes with the given Giemsa staining technique. In such case, comparisons of proteins and molecular genetic markers could still reveal a genetic introgression by *L*. *guilinensis*.

However, the allozyme comparison did not reveal any sign of *L*. *guilinensis* genome, but all analysed specimens of ‘*B*.*’ zebra* were in all of the informative proteins undistinguishable from *S*. *pulchra*. These results provide evidence that no genetic introgression by *L*. *guilinensis* has occurred.

Finally, both phylogenetic reconstructions, one on base of a mitochondrial gene and the other on base of a nuclear gene, suggested with high statistical support that ‘*B*.*’ zebra* is the sister lineage to *S*. *pulchra*, while all specimens of *Leptobotia* were only distantly related.

At the end, we report a strong discrepancy between morphological and genetic data with the former suggesting gene flow between the diploid *Leptobotia* and the tetraploid *Sinibotia* in the upper River Li basin, while the later did not reveal any sign of genetic introgression of *Leptobotia* into the evolutionary history of ‘*B*.’ *zebra*. Since the amount of information taken from the genetic analyses was high and the different methods that have been applied in the present study have analysed a wide range of genetic data (chromosomes, allozymes, mitochondrial and nuclear DNA sequences), it is very unlikely that a hybridisation event would have stayed undetected.

We finally conclude that ‘*B*.’ *zebra* is not the result of a hybridisation event, but a species of *Sinibotia* that underwent an outstanding example of evolution that has changed its morphology in the way that it strikingly matches the morphology of the co-occurring species *L guilinensis*. This seems at least surprising, since evolutionary theory pronounces that a strong selection exists against the co-occurrence of highly similar species (competitive exclusion, Gaus’s law) [37,38]. We therefore have to assume that there exists an evolutionary advantage for *S*. *zebra* in looking so similar to *L*. *guilinensis*. The most common mechanism to achieve such an advantage is mimicry; which helps to reduce the predation pressure on one (Batesian mimicry) or both (Mullerian mimicry) similar species [39,40]. Since all species of Botiidae have a suborbital spine as anti-predator weapon and six out of eight species of Botiidae in the River Li share a pattern of broad bands on the body, the possibility exists that they represent a case of Mullerian mimicry. The only species with a different pattern are *L*. *guilinensis* and *B*. *zebra*; which does not fit to the assumption of Mullerian mimicry. Nothing is known about ecology and microhabitat of *S*. *zebra* and *L*. *guilinensis*, but the their frequent occurrence in the same lot on local markets and ornamental fish imports indicate that they live very close to each other, making their case of ‘mimicry’ an interesting topic for further research.

Another result of the present study is the first record of *S*. *zebra* from outside the upper River Li basin and even outside the Pearl River basin. Specimen A8614 was found among specimens of *S*. *pulchra* from the Min River in Fujian province (Fig 1). It bears the characteristic pigmentation of *S*. *zebra* (head like *S*. *pulchra*, body like *L*. *guilinensis*) as well as the diagnostic combination of mental lobes and a simple suborbital spine and has the *S*. *zebra* specific character state of 7½ branched dorsal-fin rays. Interestingly, no species of *Leptobotia* has been recorded from this basin up to now; therefore no partner for any co-evolution as discussed above would be available. However, our phylogenetic reconstructions based on the mitochondrial and nuclear genes show that specimen A8614 from the Min basin is very closely related to their conspecifics from the Li basin. The same is true for *S*. *pulchra*; the specimens from the Min basin bear very similar haplotypes as specimens from the Li River. We consequently assume their presence in the Min basin to be the result of a very recent range extension. Range extensions along the southeaster Chinese coast were possible during the Pleistocene glacial maxima, when the lowered global sea level led to prolongation and joining of coastal rivers. However, no botiid species is known to occur on Taiwan, which also was connected to the Chinese coast during the glacial maxima in Pleistocene and therefore shares several freshwater species with the coastal rivers of China [41,42]. It is possible that the presence of botiid fishes in the Min River basin is even younger than the last glacial maximum and might be the result of human activity.
